# Lived experiences of food insecurity and mental health: the role of socio-demographic inequalities

**DOI:** 10.1186/s12889-026-28006-8

**Published:** 2026-06-10

**Authors:** Maddy Power, Madeleine Baxter

**Affiliations:** 1 Department of Public Health, Environments and Society, London School of Hygiene and Tropical Medicine, London, UK; 2 Department of Health Sciences, University of York, York, YO10 5DD UK

**Keywords:** Food insecurity, Food poverty, Mental health, Inequalities, Poverty, Ethnicity, Parenting

## Abstract

**Background:**

Food insecurity is strongly associated with mental health and also varies by demographic group but the role that socio-demography plays in shaping the relationship between food insecurity and mental health remains under-researched, particularly in the United Kingdom where food insecurity is an escalating public health emergency. This article explores how socio-demographic characteristics – specifically, income, parenting, and ethnicity – shape the lived experience of food insecurity and mental health in the UK.

**Methods:**

A diverse sample of 62 people living on a low income in England were interviewed three times over two years. Interviews addressed lived experiences of food insecurity and food aid, mental health, and experiences of discrimination. Data were analysed using reflexive thematic analysis.

**Results:**

The results suggest that the pressures of and inability to afford food may exacerbate the existing stress of navigating life on a low income, aggravating anxiety, and contributing to social exclusion and depression. Source of income shapes the nature of anxiety surrounding food, with people in receipt of welfare benefits reporting acute food insecurity and related anxiety, isolation and depression. Parents experienced heightened stress from their financial inability to provide food for their children, generating shame and precipitating parental and family isolation, which contributed to anxiety and depression. A complex matrix of structural and cultural factors increased the importance of food in the connection between financial insecurity and mental ill-health among minority ethnic participants. The high cost of ‘traditional’ foods created stress and necessitated dietary change, impacting self-identity and socialisation.

**Conclusions:**

Our findings underscore the interconnection of inequalities, food insecurity and mental health. Systemic action to address both food insecurity and mental ill-health is urgently needed; it is vital that policy and practice attends to the profound role of demographic inequalities in shaping lived experiences and outcomes.

**Supplementary Information:**

The online version contains supplementary material available at 10.1186/s12889-026-28006-8.

## Background

Food insecurity, defined as ‘limited access to nutritionally adequate food due to lack of money or other resources’ [1], is public health emergency in the United Kingdom (UK), experienced by 10% of UK households [2], and yet remains poorly understood. North American research points to striking inequalities in both the lived experience and epidemiology of food insecurity; in the UK, by contrast, little is known about socio-demographic inequalities in the prevalence and lived experience of food insecurity and how these relate to mental health. Drawing upon a longitudinal research programme with a large demographically diverse cohort in England, we address this evidence gap, exploring how socio-demographic characteristics – specifically, income, parenting, and ethnicity – shape the lived experience of food insecurity and mental health in the UK. The article documents marked inequalities in the lived experience of food insecurity, outlining how these inequalities shape the relationship between food insecurity and mental health in ways that reflect and go beyond existing evidence. We conclude by emphasising the urgent need to recognise – if not centre – inequalities in research and policy on food insecurity.

### Food insecurity and mental health

There is a well-established bi-directional relationship between food insecurity and mental ill health [3–5]. Adults living in food insecure households are more likely to experience a wide range of adverse mental health outcomes, compared to those living in food secure households, including depressive and suicidal thoughts, major depressive episodes, diagnosed mood disorders, diagnosed anxiety disorders, and self-reported poor mental health [6]. Stress is hypothesised to play a key role in the association between food insecurity and mental health; heightened levels of stress and anxiety develop from concerns surrounding accessing sufficient food [7], and existing mental health issues can be exacerbated by poor access to food, leading to a cycle of stress and deteriorating mental health [8]. Mental illness can also be caused and worsened by poor nutrition and disease consequent upon food insecurity, as well as the stress and anxiety incurred by being in a state of poor physical health [9]. Emerging UK evidence reflects findings from Canada [10] to suggest that people with severe mental illnesses, such as chronic anxiety disorders, bipolar disorder, schizophrenia, and other types of psychosis, are particularly likely to experience food insecurity [8] and, moreover, the impact of severe mental illness on daily living is predictive of food insecurity [11]. Living with a severe mental illness can compromise an individual’s ability to engage in paid employment, raising the risk of poverty, impact daily living skills such as cooking and food shopping [12], increasing the likelihood of food insecurity [11], and create additional difficulties in accessing food banks [13, 14].

### Inequalities in food insecurity and mental health

Food insecurity, like mental ill health, is not spread evenly across the UK population with income, age, disability, the presence of children in the household, and ethnicity impacting household food insecurity [15]. Emerging evidence on the relationship between food insecurity and mental health in the UK suggests a role for socio-demographic characteristics in shaping lived experiences, in particular income [16]. Income is arguably the most salient factor impacting decisions and behaviour surrounding food and eating, and there has been a marked increase in food insecurity in the UK since 2010 as incomes have been squeezed by a combination of cuts to social security payments, increased part-time work and growing job insecurity and, more recently, by rising food prices in the context of the ongoing cost-of-living crisis. Living on a low income has similarly well-established links with poor mental health [17] because the broader experience of poverty is often stressful [18]. Equally, having long-term mental ill health can precipitate a fall in income because of labour market disadvantages [19], including being unable to work due to ill health and structural barriers, such as stigma. Theoretically, the social security system should act as a safety net to protect the incomes of people living in poverty and those unable to work due to ill health, but current UK social security policies may in fact be aggravating mental ill health [20].

It is clear that parents [21, 22] and ethnic minorities [23, 24] are at higher risk of mental illness than the general population, however there is limited research specifically addressing the lived experience of food insecurity and its interaction with mental health among ethnic minorities and parents. There is a strong connection between food insecurity and parenting in the UK: in 2022-23, 25% of households with children experienced moderate, low, or very low food security, indicating difficulties accessing and affording food and, in some circumstances, hunger for adults and children; this compares to 13% of households without children [2]. An emerging body of evidence suggests that parental responsibilities can shape the relationship between food insecurity and mental ill health in the UK. The financial and emotional pressure experienced by food insecure parents in maintaining an adequate diet for their children can damage mental and emotional wellbeing, with parents reporting feelings of shame, failure and helplessness [25].

In the UK, as in North America, ethnic minorities are at increased risk of both food insecurity and mental illness compared to the white ethnic majority. Population-level surveys demonstrate ethnic inequalities in household food security, with high food insecurity among Black/African/Caribbean/Black British households and comparatively low food insecurity among White, Indian, and Chinese households [26]. People from ethnic minority groups in the UK are also more likely to have undiagnosed and untreated common mental illness [27, 28] and receive a diagnosis of severe mental illness compared to the majority white ethnic group [24]. People in ethnic minority households are more likely to be in persistent low income before and after housing costs than people in white households [29], a factor that has been positively correlated with higher rates of mental illness and food insecurity [30, 31].

The apparent raised risk of ethnic minority groups to food insecurity and the established link between food insecurity and poor mental health suggests that ethnic minority groups may be at higher risk of food insecurity-related poor mental health. Empirical evidence on the relationship between food insecurity and mental health among different ethnic groups is limited, especially in the UK. Evidence in North America indicates that food insecurity is associated with varying levels of serious psychological distress among minority ethnic populations, particularly Black/African Americans [32–34]. In the UK, quantitative analysis suggests that the relationship between food insecurity and mental health may be impacted by ethnicity [35], however the direction of this relationship is unclear with conflicting findings at the local and national level [2]. Qualitative research indicates that cultural norms, including expectations of hospitality centred around food among families and neighbours, and experiences of structural racism, may impact the lived experience of food insecurity among South Asian women [36].

## Methods

This article emerged from a longitudinal mixed methods study examining food insecurity, inequalities, and mental health in England. The qualitative fieldwork took place in three cities, two in the North of England (Bradford and York) and one in the South (London). These three cities were chosen for their different demography – York a more affluent and largely white British city, albeit with pockets of deprivation; Bradford a city of high deprivation, with a large Asian and Asian British population (32%) [37]; and London, a place of extreme wealth and income inequality and the most ethnically diverse region of the UK [38].

Participants were recruited through community groups, such as food banks and community cafes, advice centres, and snowball sampling [39]. Purposive sampling was employed to recruit a diverse sample of adults living on a low income [39], which was self-declared via the question 'are you living on a low income?'. This subjective form of income-based recruitment has been used by this team in multiple studies and has been found to be an effective method in recruiting a sample of people living on a low income while not being stigmatising [40]. Participants were not recruited on the basis of a formal assessment of their food security due to the potential for this to be experienced as stigmatising and evidence indicating that low income is a good proxy for food insecurity [41].

Recruitment materials were translated into multiple languages (Spanish, Urdu, Arabic, and Punjabi) and a minority of interviews (n = 7) were conducted with a professional translator, specifically employed for the purpose of the interview. Interviews took place between January 2023 and November 2024, with six months between each interview. Interviews lasted between 40 min and two hours, were semi-structured in format, and occurred one-to-one (plus translators where required). In total, 62 people took part in Round 1 interviews, 50 in Round 2, and 45 in Round 3, with interviews taking place in person, on the phone, and on Zoom. The sample was a diverse cohort living on a low income (Table 1). Data collection was discontinued at the point at which data saturation was reached, interpreted according to Grady [42] as the point at which: *“*new data [were] redundant of data already collected”.

**Table 1:** Sample demographics

	York	London	Bradford	Total
Gender				
Male	6	2	7	15
Female	11	14	22	47
Age				
18–24	0	0	1	1
25–34	4	4	5	13
35–44	3	6	7	16
45–54	1	3	6	10
55–64	4	2	7	13
65–74	5	1	3	9
Ethnicity				
White / White British	15	9	7	31
Mixed / multiple ethnic groups	1	0	0	1
Asian / Asian British	0	2	12	14
Black / Black British / African / Caribbean	1	4	5	10
Other ethnic group or background	0	0	5	5
Child / children in household under 18				
Yes	6	7	16	29
No	11	9	13	33
In receipt of state benefits				
Yes	11	9	12	32
No	6	7	17	30

The interview schedule (see Supplementary Information: Topic Guide), which was co-produced with people with lived experience of food insecurity and mental illness, covered multiple topics including food insecurity and food charity, mental health, access to health services, and discrimination. The interview schedules for Round 2 and 3 explored the temporality of food insecurity and mental health. While drawing upon data from the three rounds, this article does not go into detail about the temporality of food insecurity and mental health as it is addressed elsewhere [14]. The interviews were conducted by both authors with the first author conducting all Round 1 interviews and the second author conducting the majority of Round 3 interviews (due to the first author by then being on maternity leave). Where possible, we tried to match interviewers with participants so that the same interviewer conducted Rounds 2 and 3 interviews with the same participants.

All interviews were recorded and transcribed verbatim by an external company. The data were analysed inductively by both authors using thematic analysis (Braun and Clarke, 2006), informed by a theoretical framework of food insecurity, socio-demographic inequalities and mental health (outlined above). The analysis employed a three-stage process. A one-page summary provided an overview of each interview and foregrounded initial themes; participant responses to each question and for each round were summarised in a spreadsheet to enable comparison; and a thematic analysis of all data was undertaken by both authors using Nvivo 15; the authors compared emerging themes until consensus on a coding framework was reached.

The study received ethical approval from a university ethics committee and ethics were a high priority before, during, and after the interviews; the project team ensured that participants were fully aware about the nature of involvement before agreeing to take part, participants were able to stop the interview at any point, and signposting was available. All participants provided informed written consent before each interview; names are pseudonymised and any identifying characteristics changed. In recognition of their time and expertise, participants were provided with a £30 voucher for each interview.

The researchers who conducted the fieldwork (and wrote this article) do not have personal experience of food insecurity or food aid, and in this respect were ‘outsiders’ to the circumstances and experiences of participants. The longitudinal design and depth of the interviews allowed us to get to know participants as people, enhancing our capacity for empathy and understanding.

## Results

The findings are presented according to the three major socio-demographic themes of income, parenting, and ethnicity, which emerged from the data. There was necessarily considerable overlap among these socio-demographic characteristics in their intersection with food insecurity and mental ill health and, concomitantly, we consider the intersectionality of food insecurity in the Discussion.

## Income

Interviews with participants across England firmly revealed the multiple ways in which the interconnection of food insecurity and financial insecurity is detrimental to mental health. It was notable that the relationship between food insecurity and mental health was shaped by source of income – for instance, social security, employment, or pension – as well as the reliability of and the agency a participant held in respect of this income.

All participants spoke of their immense difficulties affording food, with their income insufficient to cover household expenses, recently exacerbated by rising prices amid the cost-of-living crisis. This was particularly acute for participants whose main source of income was social security, all of whom described constant difficulties managing their very low income, articulating the compromises they had to make which, for many, involved buying less food. The established link between food insecurity and mental ill-health, and the role of poverty in shaping this relationship, was clear from conversations with participants, many of whom described negative changes in their mental health consequent upon their inability to comfortably afford food and the stress integral to this. This ranged from new and increasing levels of anxiety to severe depression:

“Food insecurity, well that’s a major thing for me right now. It’s a worry. It just causes mass anxiety … I feel almost powerless to be honest. Prices are rising and I don’t really have much else to say. We have to eat. I don’t have a garden, I can’t grow my food. I’ve got limited income and I’m constantly looking for alternatives.” – Ian (Aged 55–64).

The financial inability to afford not just any food but specifically food that was considered nutritious could have a particular impact on mental health. Many participants spoke of their desire to purchase, consume and cook a healthy diet, but made clear that their financial situation precluded this, giving rise to social isolation and feelings of powerlessness and low self-esteem, and compounding existing mental illness:

“I can’t even buy in bulk because I don’t have that disposable income at hand to give me that privilege … It’s frustrating to say the least. But equally I feel … so I feel almost powerless. I can’t do much about it. I feel really low esteem … I don’t go out as much as I used to. I feel just ashamed, very much … I feel, yeah, lonely a lot of the time.” – Ian (Aged 55–64).

“Things are so expensive now in the supermarket … Everything seems to be more expensive, bread, everything … Normally I would have cooked a meal and that, but I just can’t afford to do it now … I live on sandwiches at home … I suffer [from] depression anyway … It just makes it worse don’t it?” – Deborah (Aged 65–74).

A key characteristic of food insecurity is not only difficulties affording food in the present, but concerns surrounding accessing food in the future. The longitudinal nature of our data underscored the importance of this characteristic of food insecurity in engendering chronic and long-term anxiety. Running from January 2022 to November 2024, the interviews spanned policy, political, and seasonal change. Anxieties about affording food during the cold winter months were evident in interviews conducted in spring and summer; concerns which were borne out during the Winter months when high energy costs further undermined participants’ ability to afford food:

“I’ve had to adjust in quite a lot of ways I think with food. I don’t eat as much. But I mean I’m dreading the winter coming because that’s when you need warm food … last winter was a bit grim. I don’t use my oven. I’ve got a microwave … But being summer it’s much, much easier because you’re not shivering as much. But yeah that last winter was … I always try to be positive, but it was miserable.” – Sharon (Aged 55–64, Round 1 interview).

“But I remember the first interview, after that, I don’t know whether I said anything, but my thoughts were, gosh, you know, that was a different season, and, so this time round, it’s, yes, it’s … I always try to look at the … find a silver lining, always. I can still sort of … now it’s just like a little glimmer. You know, because, yes, a lot more difficult, which I did, sort of … I mean it was inevitable really, isn’t it, because, as the seasons change and we’re coming to winter, the cold is the … when you hear people talking about choice of heating or eating, I choose to have heating on because it’s so so miserably depressing to sit in your home, and I have the loveliest home, I am so lucky and grateful. But to sit here shivering, so yes, I don’t need a lot to eat and I’d rather be warm.” – Sharon (Aged 55–64, Round 2 interview).

There is a well-established evidence base documenting the profoundly detrimental impact of social security receipt on mental health, demonstrating the stress, anxiety, and depression that is created and worsened by the inadequate and highly conditional nature of the income received, as well as the damage to mental health which can ensue from encounters within the Jobcentre [20, 43, 44]. Our data concurred with this evidence and also revealed the particular role of decisions surrounding food in the relationship between receipt of social security and mental ill health. Multiple participants in receipt of social security described the insufficiency of their income in covering their household expenses and the consequent need to prioritise housing costs and utilities over food, exacerbating anxiety and depression as they attempted and failed to manage an impossibly small budget:

“I’m on benefits, I’m on ESA [Employment Support Allowance], because I’ve got arthritis and I’ve got blood clots. I’ve got lots of problems, you know, lots of health problems . When I pay my gas bill, electric, all the other household bills, my rent, council tax, there’s nothing left for food … I’m on antidepressants, yes, with the stress . Stress, stress, stress. Stress. Just paying bills and just struggling.” – Joan (Aged 65–74).

Income from benefits could be stopped with little or no communication creating sudden destitution and, in some cases, debt. These situations created great stress and anxiety for claimants; a stress which was compounded by the additional difficulties they now experienced affording food, for many the only item in the household budget which could realistically be reduced:

“I was asking everyone what I could do, I was doing all the things I’m able to do, going on Google, phoning, knocking on doors, speaking, no, there’s no such help, no such help. I had a routine appointment at the hospital with a psychiatrist and she said I look really worried, I look really underweight, she was worried about me and I just said, “I’m really stressed. My money’s been stopped and I don’t know,” because the thing was, with the benefit system, I hadn’t even been told why my money had been stopped … You cannot not pay your rent but you can choose, use the word choose, to limit your food or change your diet.” – Helen (Aged 45–54, receives Universal Credit, Personal Independence Payment, and Employment Support Allowance).

### Parental responsibilities

Interviews with participants who were parents or carers of dependent children sharply illustrated the financial pressures of parenting and its impact on parental mental health. Household food insecurity was both a consequence of this financial pressure and a pathway between parental poverty and mental ill health. Parents in our sample routinely described the additional costs of children, especially in relation to sufficient and healthy food, and the mental health impact of stretching an already small budget, all amid the social isolation that can come from parenting. These additional costs often occurred in the context of suppressed income, either because of leaving or converting to part-time paid employment to care for children, or from a drop in income on maternity leave:

“Because I feel like … let’s be honest, healthy food is expensive, rubbish food is cheap. Having a family and being able to … they keep growing and obviously being able to eat well but sensibly … like I said you can get junk food … you can grab pizzas for what, 50p, but sometimes trying to make a healthy meal, it does cost you a bit more than having junk. So I am very conscious of it, obviously for myself and my children. But yeah I do try my utmost to try and incorporate them both, healthy and a bit of everyday food as well. So yeah it does, because I feel like it does contribute to your mental health because I think … like I said, with me being at home now, I’m not exactly where I was when I was working. If I’m at home and I’m only eating what I shouldn’t be eating, I’m going to be putting on the weight and I feel like it does add to your mental health.” – Jasmine (Aged 25–34 recently had a baby and was unable to qualify for maternity pay).

Circumstances in which parents were unable to provide adequate food for their children created not only considerable stress but a keen sense of shame, which, for some, could further parental and family isolation:

“Especially sometimes that you know where you’re worrying for things like if there is enough food to send her to school and stuff like that. And then you think if you send her, and she hasn’t got enough if they will judge or stuff … Sometimes you feel like you’re failing your kids, but it’s not your fault … But teachers don’t really see it like that, they just safeguard too quickly if you know what I mean … Whereas in my head I think as long as she goes to school and has something to eat, it might not be a lot, but at least she’s eating something, but sometimes they don’t look at it like that, they will say something … Like before the teachers have said to me is she getting fed properly? And I’m like yes but she just didn’t have enough lunch today. So, to avoid that situation when I don’t have enough lunch for her to go to school, I just keep her off.” – Gemma (Aged 25–34).

Participants without dependent children spoke of the financial pressures and anxiety that ensued from higher energy costs during Winter, and the consequent decisions to reduce food. The ‘heating or eating’ trade-off was most acute in the case of parents with dependent children, particularly those with very young children, who felt that they had little option but to keep their home warm, thereby placing extra strain on the household’s food budget and creating significant parental stress:

“Gas and electric bills are over the roof. Having to maintain that in the house, you can’t be in the house without that. Thank god now it’s getting warmer, so we don’t have to have our heating on. But this winter was a very cold winter. I had just had the baby as well, so I could not stay in a cold house. So that was one of my … that was a massive stress of mine honestly. It was difficult honestly. In the wintertime it was awful. Like I had to get help from family members and that’s something I’ve never done. I mean that was a lot for me. But I literally had to just sort of swallow my pride and just do it, because it was a lot and it was a very cold winter. I have young children as well. So I had no choice.” – Jasmine (Aged 25–34).

The additional cost of children was often at its sharpest in cases of children with health conditions; in our cohort this included parents of children with Special Educational Needs (SEN) and dietary restrictions. Parents reported stress from constantly ensuring their children avoided food which could make them unwell, as well as from the added expense of purchasing food ‘free from’ specific ingredients:

“Yes, so [my daughter] has a dairy and soya intolerance which makes everything 10 times more expensive than anything else because weirdly a lot of bread has soya in it, so you can’t buy the cheaper bread … So, it’s like £4 for a loaf of bread or something ridiculous.” – Amanda (Aged 25–34).

The pressure to purchase additional food for their children could be at its sharpest in social situations, ensuring that children were protected from the social isolation and shame associated with food insecurity:

“If I go to a birthday, I have to spend more than normal, if you know what I mean? If you take, for example, if you have your son and you need to take him to birthday, just buy the present and take him. I need to buy the present, I need to buy his cake, I need to buy his juice, just in case. I need to buy the sweets, I need to send everything with him, just to not make him feel he can’t have anything or he’s different.” – Faiza (Aged 45–54).

We described above the inadequacy of social security in protecting households from food insecurity and the negative impact of social security on mental health. It was evident from our interviews with parents that this also applied to families with children, not only in relation to the broader inadequacy of Universal Credit in covering families’ household expenses, but more specifically to the insufficiency of maternity allowance, described by Hiba, and to the failure of Free School Meals to cater to children with additional needs, placing extra pressure on the household budget, illustrated by Adam:

“I honestly think genuinely, like, it’s easier to go to work than think about food every day, literally it’s just that, like, I’m anxiety ridden that I just think it’s easier to just go to work and deal with stuff at work than having to think about what I’m going to cook, what you can afford to cook, what’s available and having to feed eight people in the household with it.” – Hiba (Aged 25–34).

“For example, separated food is something that he [his son] prefers to eat but if you are in a school kitchen and you are cooking for a selection of a hundred children or more, two hundred, I don’t know, you’re going to be mixing the sauce with the pasta as opposed to cooking plain pasta … It is stressful and it is time-consuming and we try and make it work as best as possible.” – Adam (Aged 25–34).

Adam’s experiences as a parent with a child diagnosed with autism and pica (a condition which involves eating non-food items with no nutritional value) are arguably specific and have a notably profound impact on his daily routines and household budget, creating chronic stress and anxiety. However, Adam’s experiences speak to a wider finding in which public services often failed to address the food and mental health needs of parents and their children, intensifying the financial and emotional implications of food insecurity.

### Ethnicity

Conversations with participants made clear that the relationship between food insecurity and mental health could be shaped by ethnic identity and heritage, and that the ways in which ethnicity impacted the connection between food insecurity and mental ill health operated on multiple levels. Multiple participants who described themselves as Black, Asian, or Minority Ethnic (BAME) discussed the difficulties experienced accessing foods they had grown up with and/or were used to eating, due to both availability and high cost. These challenges rendered the process of accessing food, already stressful on a low income, unenjoyable and anxiety-provoking:

“I don’t know if you’re aware of plantain, the yellow banana, that’s always in abundance. But something like yam is hard to get and it’s quite expensive as well and sometimes I think they get them, and when they get them they have to chop them up. And by the time they chop them up, a long piece of yam into like four pieces, that will probably just give you one meal for a day. Plus it’s highly perishable and expensive, so it’s not something you get in abundance. So I would say the access to that sort of food is limited, very limited, largely because of the cost … It does [impact my mental health] actually. Sometimes you’ve got to think survival mode, I’m not gonna let it bother me. It used to bother me a lot and I think I’ve just come to accept that, yeah, the sheer cost of it has a big impact on, you know, it’s not going to be able to satisfy everybody.” – Ian (Aged 55–64).

“Because they don’t sell certain foods in the supermarket, like cultural foods, like certain vegetables and stuff that you can’t get in the supermarket … like yam, breadfruit, plantain, green banana, akki, all different types of seasonings, so many I could list, and meats like oxtail, goat, mutton, things like that. Oxtail is so expensive, I don’t know if you’ve ever had oxtail before, it’s so delicious but it’s really expensive. But yes, they don’t sell that at Morrison’s or Sainsbury’s.” – Amara (Aged 35–44).

The longitudinal study design allowed for insight into the nature of price rises for some types of ‘traditional’ foods and the impact of this on the mental health of minority ethnic participants. The increasingly high and unpredictable prices of some African, Caribbean, Afro-Caribbean, and Asian food had, over the course of the three interviews, made the already-difficult process of affording food on a small budget even more stressful, and forced some participants to change their diets to incorporate less of the food that they would choose to eat:

“I come from a Caribbean background, I was UK born. But most of the food I eat is really Caribbean and Afro-Caribbean food, which now with the food crisis it’s literally shot through the roof and there’s no real alternatives. For example, like buying yams. I used to buy them regularly, but now I just can’t afford it … It’s a bit embarrassing and frustrating at times. It’s led to anxiety and stress levels going up. Because you don’t know where the prices are going to be from day to day.” – Ian (Aged 55–64, Round 3 interview).

“As I said before, I only buy things that are under a pound. And if you buy vegetables within a pound, then you don’t get fresh vegetables that much. They usually sell you the old ones for £1. The vegetables that we can get from Tesco is a different thing. I’m talking about the Asian vegetables that I need to cook for the dishes I eat. Then I have to buy from the Asian shops and these things are not like … you don’t get anything fresh within a pound much … Asian dishes, I can’t afford much of them. I used to eat them twice in a month, now I usually do it once. It’s not that easy. Everybody is suffering, we have to accept.” – Aditi (Aged 35–44, Round 3).

Aditi’s description of her increasing inability to afford the food that would compose ‘Asian’ dishes underscores the loss of agency inherent to food insecurity as food prices rise. The significant and immediate stress and anxiety borne of the financial inaccessibility of certain ‘cultural’ foods for BAME participants, was accompanied by its impact on identity and agency. For many, as well as being a source of nourishment, ‘traditional’ foods allow people to connect with family, culture, and selfhood [45]; in this way, in precluding participants from accessing the foods they would choose to eat and that allowed them to connect with family and friends, the high and rising prices of some ‘cultural’ foods, threatened self-identity and selfhood which could underpin depression:

“You open your fridge, there is no [African] food and you don’t have that much money to get what you want. It’s really tough. It’s tougher than last time we spoke … It makes me stressed out and I don’t know. It just makes me… I don’t know how to use that word, if I want to use the word paranoid. It just makes me angry and what is this? You can’t really get what you want … It does make you sad, because that is the food that you’ve been eating all your life. Not that we can’t eat food here but you just want to eat… because rice, there are different ways people make rice from their own culture. Ours, we will make our rice and what we use is a tomato stew. But if you can’t afford… you can’t just boil rice and start eating it. You need to make this stew that goes with the rice. If you can’t afford it, it can… it causes a lot of… people have depression because of it.” – Feramni (Aged 45–54).

Our data demonstrated the ways in which isolation can be a pathway between food insecurity and mental ill health as an individual avoids social interaction and engagements because of the cost pressures associated with these, or the shame of not being able to provide food for themselves and others. From conversations with participants, it was clear that the impact of food insecurity on social and familial engagement and connection was particularly pronounced for South Asian participants, for whom hosting or contributing to family meals can be a central part of life [46]. The high and rising cost of food had, for some South Asian participants, limited the frequency with which they socialised with family and friends:

“You cannot afford to have people over, friends over, family over, there’s been a massive decrease in that we can’t generally afford to eat big meals as a family, we will literally ration food and cook every four, five days and that’s it, because you can’t afford to have a fresh meal every single day, it’s just too expensive … You see them maybe once every six to eight months, like now because of this whole situation or maybe every four, five months whereas before you will be able to call people over on a Sunday, have people, family, friends over to eat but now it’s just not affordable to do that and to ask for people to contribute, they’re probably in a dire situation in comparison to yourself, so it’s just not fair.” – Hiba (Aged 25–34).

The financial inaccessibility of food thereby undermined social connection, fundamental to many communities and cultures, and particularly to South Asian cultures, eroding the protective effect of social interaction on mental health [47]. However, the cultural norm and social expectation to host and provide food for others, perceived by some South Asian participants to be especially strong within their culture, could also cause stress and depression for those unable to afford food, leading to some people effectively being self-ostracised from familial and social gatherings:

“I’m already on and up with the depression as well … I felt a little bit isolated, but you know talking about food it’s like when you have health issues and food is a big thing in our culture, so you know when you go to people they’ll have a table spread and that’s in my family, so I’m expected to do that. Even though they might not expect it but I would want to because I don’t want to be the one taking and not being able to give.” – Zainab (Aged 55–64).

Purchasing, cooking, eating, and providing food for others can be a deeply symbolic and meaningful act, a way to express one’s identity and love for others, as well as essential to celebrating and maintaining cultural heritage [48]. For BAME participants, this dimension of food was described as particularly important for their sense of selfhood and self-esteem, as well as their social and familial relationships, which were fundamentally threatened when food was unaffordable, contributing to stress, shame, anxiety, depression, and isolation.

It is well established that people with health conditions and disabilities, including mental ill health, are overrepresented at food banks [49]. Our data was rich with examples of how the racialisation of food aid could impact the mental health of BAME service users, and therefore we discuss this at length elsewhere [50]. There were some circumstances, however, in which the relationship specifically between food insecurity and mental health, and food aid use and mental health, among BAME participants, overlapped. Previous research has identified the perceived impact of cultural expectations and social ‘taboos’ on the lived experience of food insecurity among Black and South Asian women [36]. Some South Asian participants described the social taboo they experienced not only surrounding food insecurity, but also around mental health among the South Asian communities in which they lived; they were reluctant to discuss their mental health and/or seek support from family and friends, but also found there to be limited mental health support available at food banks:

“Yes, I feel like people with foodbanks and stuff and mental health, either people think you’re lazy and that’s why you’ve got mental health, or they don’t believe in mental health issues. I think especially within the Asian community there is a massive taboo that mental health doesn’t really exist, and you bring it upon yourself. So, it’s very difficult in that way. And then, like, even with foodbanks and stuff like that, when you go it’s too awkward to talk to anybody. Everyone is, kind of, just, like, already … because they’re probably feeling conscious themselves and anxious themselves, you find it uncomfortable to talk to the people about it, so you just get your things and leave and that’s it.” – Hiba (Aged 25–34).

Nevertheless, the particular difficulties that ethnic minorities may experience in accessing food only partially relate to food costs; structural racism and racial discrimination through public policy and interactions with public services can have a significant impact on the likelihood of experiencing food insecurity. This was evident in the case of Aditi, who lives in London with her young daughter and experiences significant mental health issues which severely limit her ability to work and necessitate additional support. Aditi described how her assessment for Employment Support Allowance and Personal Independence Payment was negatively impacted by mistranslation and misinterpretation:

“My English is not good enough and when a native speaker speaks fast, I don’t understand. I used an interpreter to understand their questions properly. When she said can you climb up the stairs, I said how many, she said two. Two step in Bengali, which I speak, we say two steps are the same as two stairs. She didn’t explain that to me. She said two steps. When she said I understood two steps, I said I can do two steps. I thought that I can climb two stairs without any difficulties.” – Aditi (Aged 35–44).

Aditi’s experience sharply illustrates the systemic drivers of food insecurity and its consequent impact on mental health, of which food costs is one, however, one which came through as a dominant theme in interviews. It was clear from our data that the food insecurity and mental health experiences of BAME participants could be closely shaped by their ethnic identity, in addition to their income status and parenting responsibilities. Experiences and identities were thus inherently intersectional and multifaceted, with low income, BAME mothers, for instance, often experiencing the greatest sense of exclusion consequent upon their financial status and their identity.

## Discussion and conclusions

Our analysis demonstrates the central role of socio-demography in the interconnection of food insecurity and mental health, underscoring the importance of recognising demographic differences in policy and interventions addressing both food insecurity and disabilities. It is well established that low income and mental health are intimately connected; our analysis augments understanding of the role of food in this connection. As also found in qualitative studies of food insecurity and severe mental health [11, 13], the pressures of and inability to afford food exacerbate the existing stress of navigating life on a low income, aggravating anxiety, and contributing to social exclusion and consequent loneliness and depression. Concerns affording food are acute in the present but also refer to the future, creating chronic anxiety. It was notable that the source of an individual’s income closely shaped the nature and extent of anxiety surrounding food, with people in receipt of social security for most or all of their income reporting particularly acute food insecurity and related anxiety, isolation, and depression; this reflects existing evidence on the very high prevalence of food insecurity among people in receipt of Universal Credit [2] and the detrimental mental health impacts of the social security system [51].

It is increasingly clear from population-level data that households with dependent children are at higher risk of food insecurity than those without [2]. At the same time, parental, and particularly maternal and perinatal, mental health is a major public health concern, with specialist perinatal mental health services providing care and treatment for mental health needs [52]. Our analysis identified the fundamental role of food in the mental health of parents living on a low income. Parents experienced heightened stress from their financial inability to provide food for their children, which generated feelings of shame, as also reported by Hevesi et al. [25], and precipitated parental and family isolation, which in turn contributed to anxiety and depression. With children in the household, there was less scope to reduce other household expenses, especially fuel, further squeezing food budgets. Parents of children with health conditions or additional needs experienced particular and intensified pressures with often higher costs for specialist foods and very limited support from public services in accommodating the food needs of their children, reflecting previous research [53].

It is also increasingly clear that being Black, Asian, or Minority Ethnic is a risk factor for food insecurity in the UK and yet, unlike North America, where there is a very large literature on the racialisation of food insecurity [54–56] there is real lack of evidence on the prevalence, epidemiology, and lived experience of food insecurity among BAME households in the UK. This is the first study to explain the role of ethnicity in the interconnection of food insecurity and mental health among UK households. We identified a complex matrix of structural and cultural factors which increased the importance of food in the link between financial insecurity and mental ill health. The high cost of ‘traditional’ or ‘cultural’ foods created additional stress when accessing food and had necessitated many BAME participants to change their diet, removing the food they were used to eating. This had a marked impact on self-identity and self-esteem, which was often given meaning through food, and on the ability to socialise with family and friends, contributing to social isolation. Feelings of shame borne of food insecurity could be heightened by cultural norms and expectations of providing food for others, especially prominent in South Asian cultures [46]. Beyond the elevated prices of ‘cultural’ and ‘traditional’ foods, examples of structural racism, which created and exacerbated poverty, were especially notable in the social security system, reflecting evidence on broader discrimination experienced by BAME households in accessing welfare benefits [57].

While a relatively large qualitative study, including a total of 157 interviews over two years, there are inescapable limitations. The study took place in three cities, each with their specific geographical and cultural characteristics, which will inevitably have impacted the lived experiences of participants, yet we do not discuss the impact of place here. The longitudinal nature of the study allowed for an intimacy to be established in the interviews and contributed to a richness to the data which may not have been possible from one-off interviews; nevertheless, the interview methodology has limitations both in the potentially curated presentation of experiences and the limited insight which it allows. It is also worth noting that where possible the same interviewer conducted each of the three interviews; this allowed for an intimacy and depth which may not otherwise have been possible but will inevitably have shaped how the conversation unfolded in a way that we do not critique here. We have focused here on the three socio-demographic characteristics of income, parenting, and ethnicity, which were the dominant inequalities in our data, however there are certainly other inequalities in food insecurity, most notably age and geography, which will shape experiences and warrant consideration and analysis.

Food insecurity is a public health crisis in the UK *because* it is a strong predictor and consequence of mental and physical ill health – with ever-rising household food insecurity we are likely to see further pressures on mental and physical health services, as well as increasing numbers of people with disabilities attending food banks. There is arguably both a moral imperative to address rising household food insecurity in the UK – the social injustice of any adult or child being unable to afford sufficient food – and also a policy imperative in the impact of food insecurity on public services [58]. The cost of disability benefits has risen sharply in the UK in the aftermath of the COVID-19 pandemic and, as such, is high on the policy agenda of the Labour government. Our study arguably opens more questions than it answers, and we present it as a stepping stone in an essential move towards broader and deeper analysis of the role of inequalities in food insecurity and mental health in the UK.

## Supplementary Information

Supplementary Material 1.

## Data Availability

The datasets generated and/or analysed during the current study are not publicly available due to ongoing publications based on the data but are available from the corresponding author on reasonable request.
