# Supplementary material for: Lived experiences of food insecurity and mental health: the role of socio-demographic inequalities
Source: BMC Public Health. 2026 Jun 10;26:2365. doi: 10.1186/s12889-026-28006-8 (PMC13474710; doi:10.1186/s12889-026-28006-8)
Supplement: Supplementary file 1 [file 12889_2026_28006_MOESM1_ESM.docx]

**TOPIC GUIDE: Food for Thought: Ethnic variations in food insecurity and mental health**

**Introduction**

Check if interviewee has any questions about the participant information sheet, consent form or interview itself. Ask if they want a brief overview of the study.

Ask them for written or oral consent.

Remind participant that they can stop the interview at any time, without giving a reason and they will still be paid for their time. Remind that the interview will be audio recorded.

Check the person has details to hand of a person they can contact for support if they should feel distressed at any point. Information about support services can also be provided by the researcher.

Outline boundaries of confidentiality. In the case of any disclosure of harm to self or others, interview will be paused, support to be offered and relevant individuals/agencies may be informed.

General

Can you tell me a bit about yourself – for instance, where you live and who you live with, what you’re up to at the moment.

Food

**First of all, I would like to chat a bit about food – about accessing food and about any barriers to accessing food.**

- Can you access the food you would like to? Why not? Past experiences? People you know?
- Have you come across the terms ‘food poverty’ or ‘food insecurity’?

Income and employment

**I want to talk a bit about money and finances, how this can affect your mental health and what makes things better – please do say what only what you are comfortable saying and you don’t have to answer any questions if you don’t want to.**

- Compared to three months ago, **how would you say you are doing financially right now**? How has this impacted on your ability to access food?
- **Impact on your mental health** or wellbeing (positively or negatively)?
- Have you tried to seek **support for any financial difficulties in the past?**
- Anything that has been **helpful in accessing the food you need?**
- What support do you **think would be helpful** for people financially?

Food banks

**We know that people are finding it increasingly hard to get enough food because we see more and more charities providing help with food in London. Why do you think that this may be happening?**

- Do you know about **places in the community where you can get food** at no or low cost, like food banks?
- What might it like to be to **go to a food bank**?
- **Do you know anyone** who goes? **Do you go?**
- **Where else** might someone get support?
- Have you accessed **support in other ways** – like family, friends or voluntary organisations? What was your experience of accessing this support?

Mental health

**I want to talk next a bit about mental health and wellbeing but please do say if there are any questions you would rather not answer.**

- There is clearly a big connection between the food you eat and your mental health and wellbeing.
- **Do you worry about your mental health?**
- Does food and do the decisions **around food cause you any stress or unhappiness?**
- Many people access mental health service because they feel low or worried. **Have you ever accessed mental health services?**
- Have you accessed **support for your mental health in other ways**, such as, family, friends or voluntary organisations? What was your experience of accessing this support?

Finally…

- What might be the **things that would make a difference** to being able to provide the food you want?
- What might be the **solutions** to these problems that (you and) others experience?
- Prompts: Income, regular employment, cost of food, cost and ease of getting to the shops, physical cooking space, social context and high price of local shops, less discrimination and stigma, support services.

**Close of interview**

Wellbeing check and offer signposting if required. Check how it was for them on Zoom.

Ask whether they would like to be paid in cash or a voucher

Discuss follow-up interview

Ask if they know anyone else who might like to be interviewed

Inform key worker/support worker
